# Eight pharmacokinetic genetic variants are not associated with the risk of bleeding from direct oral anticoagulants in non-valvular atrial fibrillation patients

**DOI:** 10.3389/fphar.2022.1007113

**Published:** 2022-11-24

**Authors:** Alessandra M. Campos-Staffico, Michael P. Dorsch, Geoffrey D. Barnes, Hao-Jie Zhu, Nita A. Limdi, Jasmine A. Luzum

**Affiliations:** ^1^ Department of Clinical Pharmacy, College of Pharmacy, University of Michigan, Ann Arbor, MI, United States; ^2^ Division of Cardiovascular Medicine, Department of Internal Medicine, University of Michigan, Ann Arbor, MI, United States; ^3^ Department of Neurology, School of Medicine, University of Alabama at Birmingham, Birmingham, AL, United States

**Keywords:** atrial fibrillation, bleeding, anticoagulation, pharmacogenetics, DOAC

## Abstract

**Background:** Atrial fibrillation (AF) is the leading cause of ischemic stroke and treatment has focused on reducing this risk through anticoagulation. Direct Oral Anticoagulants (DOACs) are the first-line guideline-recommended therapy since they are as effective and overall safer than warfarin in preventing AF-related stroke. Although patients bleed less from DOACs compared to warfarin, bleeding remains the primary safety concern with this therapy.

**Hypothesis:** Genetic variants known to modify the function of metabolic enzymes or transporters involved in the pharmacokinetics (PK) of DOACs could increase the risk of bleeding.

**Aim:** To assess the association of eight, functional PK-related single nucleotide variants (SNVs) in five genes (*ABCB1*, *ABCG2*, *CYP2J2*, *CYP3A4*, *CYP3A5*) with the risk of bleeding from DOACs in non-valvular AF patients.

**Methods:** A retrospective cohort study was carried out with 2,364 self-identified white non-valvular AF patients treated with either rivaroxaban or apixaban. Genotyping was performed with Illumina Infinium CoreExome v12.1 bead arrays by the Michigan Genomics Initiative biobank. The primary endpoint was a composite of major and clinically relevant non-major bleeding. Cox proportional hazards regression with time-varying analysis assessed the association of the eight PK-related SNVs with the risk of bleeding from DOACs in unadjusted and covariate-adjusted models. The pre-specified primary analysis was the covariate-adjusted, additive genetic models. Six tests were performed in the primary analysis as three SNVs are in the same haplotype, and thus *p*-values below the Bonferroni-corrected level of 8.33e-3 were considered statistically significant.

**Results:** In the primary analysis, none of the SNVs met the Bonferroni-corrected level of statistical significance (all *p* > 0.1). In exploratory analyses with other genetic models, the *ABCB1* (rs4148732) GG genotype tended to be associated with the risk of bleeding from rivaroxaban [HR: 1.391 (95%CI: 1.019–1.900); *p* = 0.038] but not from apixaban (*p* = 0.487).

**Conclusion:** Eight functional PK-related genetic variants were not significantly associated with bleeding from either rivaroxaban or apixaban in more than 2,000 AF self-identified white outpatients.

## Graphical Abstract

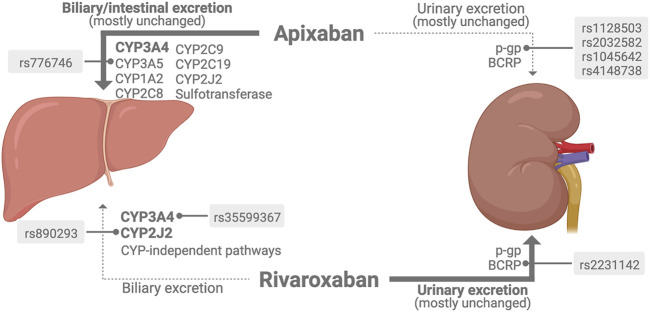

## Introduction

Atrial fibrillation (AF) is the most common cardiac arrhythmia worldwide and the leading cause of ischemic stroke (Virani et al., 2020). In the US, AF causes about 465,000 hospitalizations (Virani et al., 2020) and 158,000 deaths yearly (Centers for Disease Control and Prevention, 2020), and accounts for up to 20.4% of all ischemic strokes (Alkhouli et al., 2018). Treatment of AF is largely focused on preventing cerebral ischemia through the use of anticoagulant drugs, which reduces the risk of stroke by 66% (Hart et al., 2007). Currently, direct oral anticoagulants (DOACs) are the guideline-recommended first-line therapy (January et al., 2019) since these drugs have proven to be at least equally effective as warfarin in preventing AF-related stroke (Van Ganse et al., 2020). Although DOACs are more convenient and considered safer than warfarin overall (Wang et al., 2020), bleeding still is the main safety concern of this long-term oral therapy. Over 6 years, emergency department (ED) visits for DOAC-related bleeding jumped from 2.3% to 37.9% with the two most prescribed DOACs in the US: rivaroxaban and apixaban (Geller et al., 2020).

Rivaroxaban and apixaban have in common the direct and highly selective inhibition of factor Xa. However, there are subtle pharmacokinetic (PK) differences between these two DOACs (see Graphical Abstract). Two-thirds of rivaroxaban is metabolized by the liver through CYP3A4 (18%), CYP2J2 (14%), and CYP-independent mechanisms (Mueck et al., 2013, 2014). A third of non-metabolized rivaroxaban is renally excreted *via* the efflux transporters p-glycoprotein (p-gp) – encoded by *ABCB1* gene – and breast cancer resistance protein (BCRP) – encoded by *ABCG2* gene (Gnoth et al., 2011; Mueck et al., 2013). In contrast, apixaban is primarily metabolized by CYP3A4/5 with minor contributions from CYP1A2, CYP2C8, CYP2C9, CYP2C19, and CYP2J2 isoenzymes (Wang et al., 2010), and secondly by sulfotransferase (Wang et al., 2009). Apixaban is also a substrate of both p-gp and BCRP efflux transporters (Zhang et al., 2013) and is mainly excreted in feces (47%) and urine (29%) (Byon et al., 2019).

Genetic variants known to modify the function of metabolic enzymes or transporters involved in the pharmacokinetics (PK) of DOACs could hypothetically increase the risk of bleeding from these anticoagulants (Kuehl et al., 2001; Salama et al., 2006; Tomlinson et al., 2010; Wang et al., 2011). For example, the 6981A>G (rs776746) single nucleotide variant (SNV) in *CYP3A5* (CYP3A5*3) causes a splicing defect that results in complete loss of enzyme function (Kuehl et al., 2001; Pharmacogene Variation Consortium (PharmVar)). The effect of genetic variants on the PK of DOACs is being increasingly investigated in various pharmacogenetic studies (a summary of previous pharmacogenetic studies of DOACs is displayed in Supplementary Table S1), but the evidence is still unclear. Most of the previous pharmacogenetic studies in this area only focused on changes in plasma concentrations of DOACs (Dimatteo et al., 2016; Ueshima et al., 2017, 2018; Cosmi et al., 2019), and thus it is still unknown whether that translates into differences in the clinical bleeding outcomes.

The few pharmacogenetic studies that used bleeding as an outcome have small sample sizes (typically n < 400), which may have led to their inconclusive results (Ing Lorenzini et al., 2016; Sennesael et al., 2018; Roşian et al., 2020b). The largest pharmacogenetic study performed to date with 1,806 Finnish patients found the *ABCB1* (rs4148738) GG genotype as a protective factor for bleeding from apixaban if compared to A carriers (Lähteenmäki et al., 2021). However, those results have not yet been reproduced in other clinical studies. Therefore, this study aims to assess the association of eight functional PK-related genetic variants and the risk of bleeding from DOACs in a large sample of patients diagnosed with non-valvular AF treated with either rivaroxaban or apixaban.

## Materials and methods

### Study design and cohort identification

This single-center retrospective cohort study was carried out with 2,364 white outpatients diagnosed with non-valvular AF undergoing treatment with either rivaroxaban or apixaban. Outpatients at Michigan Medicine – a large academic healthcare system affiliated with the University of Michigan in the United States – with atrial fibrillation (n = 86,495) were initially identified according to a validated algorithm (Vanderbilt University PheKB, 2012) through a self-serve tool (DataDirect) that enables the automated data collection from the Electronic Health Record (EHR).

**FIGURE 1:**
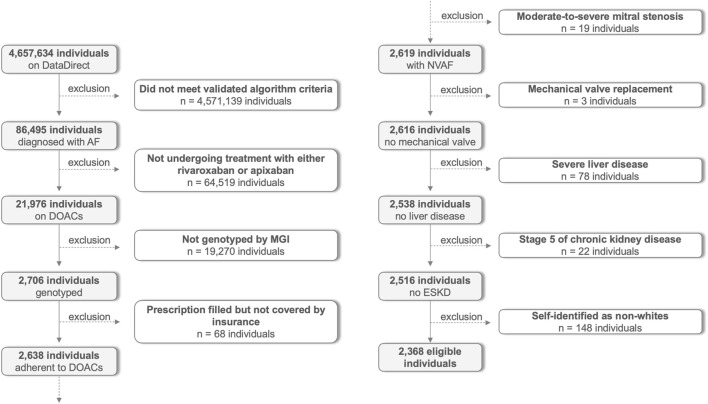
Flow chart with participants’ selection. AF: Atrial fibrillation; DOACs: Direct oral anticoagulants; MGI: Michigan Genomics Initiative; NVAF: Non-valvular atrial fibrillation; ESKD: End-stage kidney disease.

After automated identification based on the diagnosis of AF with subsequent prescription of rivaroxaban or apixaban, each individual was screened for inclusion and exclusion criteria. The complete flow chart showing the participants’ selection is found in Figure 1. Eligible self-identified white and genotyped patients aged 18 or older undergoing treatment for non-valvular AF either with rivaroxaban or apixaban were carefully selected between 1 October 2012 and 31 August 2022. Participants were excluded if they: 1) were diagnosed with moderate-to-severe mitral stenosis; 2) had a history of mechanical valve replacement; 3) were in stage 5 of chronic kidney disease defined as a creatinine clearance of less than 15 mL per minute estimated by the Cockcroft-Gault equation (Cockcroft and Gault, 1976); 4) required renal replacement therapy; 5) were diagnosed with liver disease including those with non-alcoholic fatty liver disease, cirrhosis, total bilirubin >2× normal with AST/ALT/AP > 3× normal, or other severe liver impairment as noted by the physician; 6) were not routinely followed-up by Michigan Medicine; and 7) did not have genotype data available through the Michigan Genomics Initiative (MGI) biobank (University of Michigan, 2022). The study was carried out in accordance with the Declaration of Helsinki and was approved by the local Institutional Review Board with a waiver of informed consent.

### Clinical and biochemical evaluation

All clinical and biochemical data for eligible participants were obtained from the University of Michigan data warehouse and completed by trained investigators, as previously described (Hanigan et al., 2020). The first day of the study (index date) was set as the start date of DOAC therapy. Briefly, complete biochemical and clinical summaries were performed through comorbidities identified from the participant’s active problem list or keyword note search at the baseline – defined as the period up to 1 year before the index date. The duration of DOAC treatment was determined through prescription history and keyword search using EMERSE (Hanauer et al., 2015). Study data were collected through a standardized method using a customized form using REDCap (Harris et al., 2009, 2019), and electronic data capture tools are hosted at the University of Michigan. CHA_2_DS_2_-VASc, HAS-BLED, and ATRIA scores were calculated as previously described (Lip et al., 2010; Pisters et al., 2010; Fang et al., 2011). Elixhauser comorbidities scores were computed using the “icd” package for R (Wasey JO, 2018).

### Study endpoints and adjudication of events

The primary endpoint of this study was a composite of major and clinically relevant non-major (CRNM) bleeding according to the International Society on Thrombosis and Haemostasis (ISTH) criteria (Schulman et al., 2010; Kaatz et al., 2015). The bleeding event must have occurred within the start and stop dates of the DOAC prescription. Briefly, major bleeding was defined as clinically overt non-surgical bleeding with the symptomatic presentation and 1) fatal outcome; and/or 2) involvement of critical anatomical area or site such as intracranial, spinal, intraocular followed by vision changes, pericardial, articular, retroperitoneal, intramuscular with compartment syndrome; and/or 3) hemoglobin fall fall of 2 g/dl or more, or leading to transfusion of two or more units of whole blood or red cells. CRNM bleeding was defined as overt bleeding that does not meet major bleeding criteria but requires at least one of the following criteria: 1) medical intervention by a healthcare professional; and/or 2) hospitalization or increased level of care; and/or 3) a face-to-face evaluation. Since the ISTH are strictly clinical criteria and there is no validated algorithm to help electronically identify bleeding cases according to these criteria, bleeding events were adjudicated and classified by trained clinicians who reviewed all participants’ medical notes using EMERSE (Hanauer et al., 2015). Participants were censored at the time of the first CRNM or major bleeding event or at the end of the study (the date when medical notes were last reviewed). Follow-up time was calculated as the difference between censoring and index dates.

### Exposure to and drug interaction with DOACs

Normalized daily doses of rivaroxaban and apixaban were calculated by multiplying the prescribed strength by the number of daily doses (usually one for rivaroxaban and two for apixaban) and then divided by the daily maintenance dose of each drug (20 mg for rivaroxaban and 10 mg for apixaban). Time-varying exposure to DOACs was calculated as the normalized dose multiplied by the duration of treatment within each treatment period (i.e., prescription start and stop dates). Cumulative exposure was the sum of all time-varying exposure over the follow-up period. Drug-drug interactions with DOACs were defined as the concomitant and systemic use of either CYP/p-gp inhibitors or inducers with good or excellent documentation of evidence on Micromedex (IBM Micromedex Solutions, 2022). Twelve drugs were considered as CYP/p-gp inhibitors 1) amiodarone, 2) clarithromycin, 3) conivaptan, 4) cyclosporine, 5) diltiazem, 6) dronedarone, 7) fluconazole, 8) itraconazole, 9) ritonavir, 10) erythromycin, 11) ketoconazole, and 12) verapamil; and seven drugs were considered as CYP/p-gp inducers 1) apalutamide, 2) carbamazepine, 3) fosphenytoin, 4) nevirapine, 5) oxcarbazepine, 6) phenytoin, and 7) rifampin for drug interactions with DOACs.

### Candidate variant selection and genotyping

Genes involved in the PK properties of rivaroxaban and apixaban were considered potential candidates. PubMed and the Pharmacogenomics Knowledgebase (PharmGKB) were searched in December 2021 using the following terms [(polymorphism OR genetic variant) AND (drug response OR pharmacogenetic effect) AND (gene)]. Candidate genetic variants were selected according to the following criteria: 1) minor allele frequency (MAF) ≥5% in European ancestry; 2) variants with clinical and/or functional effects; and 3) genetic variants that have been imputed or genotyped by Michigan Genomics Initiative and whose information is part of our genomic bank.

Genomic DNA was extracted from peripheral blood leukocytes and genotyping was performed as part of the MGI, as previously described elsewhere (Fritsche et al., 2018, 2019). Briefly, samples were genotyped at the University of Michigan Advanced Genomics Core lab using Illumina Infinium CoreExome v12.1 bead arrays^®^ (Illumina, San Diego, CA) with standard quality checks (Zajac et al., 2019). Eight functional single nucleotide variants (SNVs) distributed in five different genes encoding either metabolic enzymes or transporters related to the PK of DOACs were chosen for analysis. The PK properties of DOACs mapping the eight SNVs were illustrated using BioRender (BioRender, 2022) and are found in the Graphical Abstract. The eight selected SNVs are as follows: three *ABCB1* SNVs located in exons 13 (rs1128503, c.1236 T>C, p. Gly412Gly), 22 (rs2032582, c.2677T>G/A, p. Ser893Thr/Ala) and 26 (rs1045642, c.3435T>C, p. Ile1145Ile); one *ABCB1* SNV in intron 18 (rs4148738, c.2482–2236G>A); one *ABCG2* SNV in exon 5 (rs22131142, c.421C>A, p. Glu141Lys); one *CYP3A4* SNV in intron 6 (rs35599367, c.15389C>T); one *CYP3A5* SNV in intron 3 (rs776746, c.6981A>G); and one *CYP2J2* SNV in the gene promoter (rs890293, c.-76G>T). All eight SNVs were imputed. Genotype imputation was performed by the MGI using the Michigan Imputation Served and Trans-Omics for Precision Medicine (TOPMed) reference panel, resulting in ∼52 million variants (post quality control filtering) with imputation *r* ^2^ > 0.3 (Taliun et al., 2021).

Since the three closely positioned *ABCB1* SNVs – 1236T>C (rs1128503), 2677T>G/A (rs2032582), and 3435T>C (rs1045642) – are in high linkage disequilibrium, these three SNVs were analyzed as diplotypes. As 1236C-2677G-3435C is considered the wild-type, three diplotype subgroups encoding an additive 0/1/2 modeling were created as follows: 1) homozygous: 1236C-2677G-3435C/1236C-2677G-3435C; 2) heterozygous: 1236C-2677G-3435C/haplotype other than 1236C-2677G-3435C; and 3) other: haplotype other than 1236C-2677G-3435C/haplotype other than 1236C-2677G-3435C. Haplotypes were estimated from the genotype data by using the PHASE (version 2.1.1) software package.

### Statistical analysis

Data distribution was assessed for normality using the Kolmogorov-Smirnov test and visual inspection of plots. Normally distributed baseline data are presented as mean ± SD and non-normally distributed data are presented as the median and interquartile range (IQR). Categorical and continuous baseline data were tested using chi-square and Student t-tests, respectively. Hardy-Weinberg Equilibrium was assessed using the chi-square test. Participants were stratified into two subgroups according to their bleeding status. Multivariable Cox proportional hazards regression models with time-varying analysis were used to assess independent clinical predictors for bleeding risk from either rivaroxaban or apixaban (those with *p* < 0.05). Then, Cox proportional hazards regression models with time-varying analysis were used again to assess the association of each SNV with the risk of bleeding from DOACs with the significant clinical predictors used as covariates in model adjustment for the SNVs. Hazard ratios (HR) and respective 95% confidence intervals (95%CI) were calculated for unadjusted (Model 1) and adjusted models (Model 2). The primary analysis was pre-specified as the clinical covariate-adjusted models (Model 2) in the additive genetic model for each SNV (i.e., six statistical tests for the primary SNV analysis because three of the eight SNVs are in the same haplotype). Exploratory analyses were performed to assess the association of other genetic models (i.e., dominant and recessive), each *ABCB1* haplotype, and for rivaroxaban and apixaban separately. *p*-values below the Bonferroni-corrected level of 8.33 × 10^−3^ were considered statistically significant for the primary SNV analyses. This primary analysis had an estimated power of 80% to detect an HR of ≥1.26 for each variant. RGui was used for all statistical analyses.

## Results

### Baseline characteristics

The clinical and biochemical baseline characteristics of participants stratified by composite endpoint are shown in Table 1. Participants who bled with DOACs were older, had higher rates of previous surgery or trauma, transcatheter aortic valve replacement, previous bleeds, prior thrombosis, former smokers, and higher CHA_2_DS_2_-VASc, HAS-BLED, ATRIA, and Elixhauser comorbidities scores than the participants who did not bleed. There was no significant difference in any of the other variables assessed, such as sex, ethnicity, previous stroke, transient ischemic attack, body mass index, blood pressure, creatinine clearance, platelet count, hemoglobin, or follow-up time.

**TABLE 1:** Clinical and biochemical baseline characteristics of participants.

Characteristics	Overall	CRNM + major bleeding		*p*-value
		No	Yes	
Self-identified white participants, n (%)	2364 (100.0)	1952 (82.6)	412 (17.4)	—
Age, years	68.3 (13.6)	67.7 (13.8)	71.0 (12.4)	<0.001
Sex, n (%)				0.553
** **Female	758 (32.1)	631 (32.3)	127 (30.8)	
** **Male	1606 (67.9)	1321 (67.7)	285 (69.2)	
Ethnicity, n (%)				0.553
** **Non-Hispanic or Latino	2312 (97.8)	1911 (97.9)	401 (97.3)	
** **Hispanic or Latino	10 (0.4)	7 (0.4)	3 (0.7)	
** **Unknown	42 (1.8)	34 (1.7)	8 (1.9)	
DOAC, n (%)				<0.001
** **Rivaroxaban	802 (33.9)	654 (33.5)	148 (35.9)	
** **Apixaban	1324 (56.0)	1124 (57.6)	200 (48.6)	
** **Both DOACs not simultaneously	238 (10.1)	174 (8.9)	64 (15.5)	
Daily dose of DOAC, mg				
Rivaroxaban	19.3 ± 3.5	19.3 ± 3.6	19.3 ± 2.3	0.897
Apixaban	7.8 ± 4.1	7.6 ± 4.2	9.1 ± 2.6	<0.001
Cumulative dose of DOAC, g				
Rivaroxaban	17.2 ± 15.5	16.4 ± 15.2	21.0 ± 17.0	<0.001
Apixaban	7.9 ± 6.5	7.5 ± 6.4	9.7 ± 7.0	<0.001
Drug-drug interactions with DOACs, n (%)				
CYP/p-gp inhibitors	1046 (44.5)	807 (41.3)	239 (58.0)	<0.001
CYP/p-gp inducers	33 (1.4)	24 (1.2)	9 (2.2)	0.133
Previous surgery or trauma, n (%)	1449 (61.3)	1162 (59.5)	287 (69.7)	<0.001
Transcatheter aortic valve replacement, n (%)	22 (0.9)	14 (0.7)	8 (1.9)	0.019
Previous bleeding, n (%)	491 (20.8)	384 (19.7)	107 (26.0)	0.005
Previous stroke, n (%)	50 (2.1)	37 (1.9)	13 (3.2)	0.106
Previous transient ischemic attack, n (%)	37 (1.6)	30 (1.5)	7 (1.7)	0.810
Previous thromboembolism, n (%)	90 (3.8)	65 (3.3)	25 (6.1)	0.008
CHA_2_DS_2_-VASc score	2.4 ± 1.5	2.4 ± 1.5	2.8 ± 1.6	<0.001
Elixhauser comorbidities score	11.0 ± 11.3	10.7 ± 11.3	12.4 ± 11.1	<0.001
HAS-BLED score	1.3 ± 1.1	1.2 ± 1.0	1.5 ± 1.1	<0.001
ATRIA score	2.3 ± 2.0	2.2 ± 2.0	2.7 ± 2.0	0.002
Smoking habit, n (%)				<0.001
Never	1052 (44.5)	896 (45.9)	156 (37.9)	
Current	67 (2.8)	61 (3.1)	6 (1.5)	
Former	1222 (51.7)	972 (49.8)	250 (60.7)	
Unknown	23 (1.0)	23 (1.2)	0 (0.0)	
Body mass index, kg/m^2^	29.9 (8.4)	30.0 (8.4)	29.8 (8.8)	0.715
Systolic blood pressure, mmHg	127.5 (21.5)	127.5 (22.0)	128.0 (19.5)	0.350
Diastolic blood pressure, mmHg	71.0 (11.5)	71.0 (11.5)	70.0 (10.3)	0.174
Creatinine clearance, mL/min	92.2 (49.0)	92.6 (48.8)	89.7 (50.6)	0.248
Platelet count, 10^9^/L	209.8 (74.0)	209.0 (71.0)	212.5 (89.3)	0.069
Hemoglobin, g/dL	13.5 (2.5)	13.5 (2.4)	13.3 (2.6)	0.388
Follow-up time, (days)	828.2 ± 739.8	834.2 ± 740.9	800.2 ± 734.5	0.397

CRNM: clinically relevant non-major; DOAC: direct oral anticoagulants; CYP: Cytochrome P450; p-gp: p-glycoprotein.

Additionally, the clinical and biochemical baseline characteristics of participants stratified by major bleeding and by DOAC treatment are shown in Supplementary Tables S2, S3, respectively. Briefly, patients who had major bleeding were older, changed from one to another DOAC more frequently, and were significantly more exposed to CYP/p-gp inhibitors while on DOACs than their counterparts. Also, they underwent more significant transcatheter aortic valve replacement and had higher CHA_2_DS_2_-VASc, HAS-BLED, ATRIA, and Elixhauser comorbidities scores than those who did not bleed. A significantly higher frequency of former smokers and lower diastolic blood pressure, creatinine clearance, and hemoglobin levels at baseline was observed among patients who majorly bled than their counterparts (Supplementary Table S2).

Regarding patients on rivaroxaban, they were significantly healthier than patients on apixaban. Although patients on rivaroxaban bled more frequently than their counterparts, they were significantly younger, had less frequency of surgery or trauma, transcatheter aortic valve replacement, previous bleeding, and stroke, had significantly lower CHA_2_DS_2_-VASc, HAS-BLED, ATRIA and Elixhauser comorbidities scores and higher creatinine clearance than those on apixaban. In contrast, patients on rivaroxaban had significantly higher body mass index, were more frequently exposed to CYP/p-gp inhibitors while anticoagulated and were followed up for a longer time than their counterparts (Supplementary Table S3).

### DOAC treatment and prevalence and incidence of bleeding

A total of 802 participants (33.9%) were exclusively treated with rivaroxaban, 1,324 participants (56.0%) were exclusively treated with apixaban, and 238 participants (10.1%) started anticoagulation with one of the two DOACs and then switched to the other DOAC. The two main causes observed for this change during the medical chart reviews were: 1) non-coverage of the DOAC initially prescribed by the health insurance (affordability), and 2) bleeding.

A total of 412 CRNM and major bleeding events were observed in unique patients with a prevalence of 17.4% at a mean follow-up time of 2.3 ± 2.0 years (828.2 ± 739.8 days). Of these 412 patients who bled, 35 of them (8.5%) recurred either major or CRNM bleeding. The prevalence of bleeding was significantly higher among the 1,040 participants treated with rivaroxaban than among those 1,562 participants treated with apixaban (17.69% *versus* 14.59%, *p* = 0.034, respectively). The overall incidence was 7.25 bleeding events per 100 person-years, being significantly higher among participants treated with rivaroxaban than apixaban [8.32 (95%CI: 7.16–9.61) *vs.* 6.57 (95%CI: 5.74–7.48) bleeding events per 100 person-years; *p*-value = 0.018, respectively], and resulting in an incidence rate ratio of 1.27 (95%CI: 1.04–1.54) bleeding events per 100 person-years for rivaroxaban *versus* apixaban.

Although there was no difference in the follow-up time between the bleeding groups, the cumulative dose of both rivaroxaban and apixaban were significantly higher among participants who bled in comparison to those who did not bleed (Rivaroxaban: 21.02 ± 16.95 *versus* 16.38 ± 15.18 g, *p* < 0.001. Apixaban: 9.70 ± 7.01 *versus* 7.48 ± 6.39 g, *p* < 0.001). The daily dose of apixaban was significantly higher in those who bled than their counterparts (9.09 ± 2.62 *versus* 7.60 ± 4.20 mg, *p* < 0.001), and there was no significant difference in the daily doses of rivaroxaban between bleeding groups (19.30 ± 2.35 *versus* 19.27 ± 3.61 mg, *p* = 0.897). Regarding drug-drug interactions with DOACs, concomitant use of CYP/p-gp inhibitors was significantly higher among participants who bled compared to those who did not bleed (239 (58.0%) versus 807 (41.3%), *p* < 0.001, respectively). However, there was no significant difference in the concomitant use of CYP/p-gp inducers between the study groups (24 (1.2%) versus 9 (2.2%), *p* = 0.133).

### Independent clinical predictors of bleeding from DOACs

Clinical and biochemical variables that either differed at baseline or varied over the follow-up time were tested using multivariable Cox proportional hazards regression with time-varying analysis to identify independent clinical predictors of major + CRNM bleeding from DOACs in our patient sample. The results are shown in Table 2. Age, previous bleeding, Elixhauser comorbidities score, previous thromboembolism, former and current smoking, use of rivaroxaban (*vs.* apixaban), and normalized dose of DOAC were independently associated with the study endpoint and were selected as covariates for the SNV analyses. After adjustment for clinical predictors, compared to apixaban users, rivaroxaban users were at a 33% higher risk of bleeding (*p* = 0.003).

**TABLE 2:** Multivariable Cox proportional hazards regression with time-varying analysis assessing clinical characteristics as clinical predictors of major + CRNM bleeding from DOACs.

	β	SE (β)	P	eβ (HR)	95% CI for HR	
					Lower	Upper
Age, years	0.024	0.006	<0.001	1.025	1.012	1.037
History of surgery or trauma	0.067	0.116	0.585	1.069	0.842	1.359
Previous bleeding	0.346	0.122	0.005	1.413	1.110	1.799
CHA_2_DS_2_-VASc score	0.064	0.042	0.133	1.066	0.981	1.158
Elixhauser comorbidities score	0.016	0.005	0.002	1.016	1.006	1.026
Previous thromboembolism	0.484	0.230	0.042	1.623	1.019	2.586
Former and current smoking	0.122	0.049	0.016	1.130	1.023	1.248
DOAC (apixaban = 0; rivaroxaban = 1)	0.289	0.097	0.003	1.335	1.105	1.613
Normalized dose of DOAC, %	0.007	0.002	0.006	1.007	1.002	1.012
Concomitant use of CYP/p-gp inhibitors	0.099	0.124	0.447	1.105	0.855	1.428
Creatinine clearance, mL/min	1.768e^−05^	0.002	0.991	1.000	0.997	1.003

DOAC: direct oral anticoagulants; CYP: Cytochrome P450; p-gp: p-glycoprotein; HR: hazard ratio; 95%CI: 95% confidence interval.

### PK-related genetic variants and the risk of bleeding from DOACs

The genotype and allele frequencies of the eight genetic variants are shown in Table 3. All genotype frequencies were in Hardy-Weinberg equilibrium with *p*-values >0.05, and all of the allele frequencies were similar to those previously reported for Europeans in publicly available databases (i.e., Ensembl, gnomAD) or previous publications. In the pre-specified primary analysis (i.e., clinical covariate-adjusted Model 2 with the additive genetic model), none of the SNVs met the Bonferroni-corrected level of statistical significance (Table 4). In exploratory analyses with the dominant and recessive genetic models, some of the tests tended to show an association for SNVs in *CYP3A5* (rs776746) and *ABCB1* (rs4148732) (Supplementary Table S4). Also, another exploratory analysis was performed to scrutinize the association of PK-related genetic variants with bleeding from rivaroxaban and apixaban separately (Supplementary Table S5). Similar to the results for the two DOACs combined, none of the variants were statistically significant in the additive genetic model, and the associations with *CYP3A5* (rs776746) and *ABCB1* (rs4148732) in other genetic models seemed to be stronger for rivaroxaban than apixaban. The *ABCB1* (rs4148732) GG genotype tended to be associated with a higher risk of bleeding from rivaroxaban [HR: 1.39 (95%CI: 1.02–1.90; *p* = 0.038] but not from apixaban (*p* = 0.487). Since the *ABCB1* diplotypes were not significantly associated with bleeding from DOACs, an exploratory analysis scrutinizing the association of each *ABCB1* haplotype with the risk of bleeding was performed, and the results are shown in Table 5. The 1236C-2677G-3435T *ABCB1* haplotype tended to be protective against bleeding (*p* = 0.036).

**TABLE 3:** Genotype and allele frequency, and Hardy-Weinberg equilibrium (HWE) assessment of the eight PK-related genetic variants.

Allele frequency		Statistical comparison of allele frequency (χ^2^; *p*-value)	Genotypes and frequencies, n (%)	HWE (χ^2^; *p*-value)
European ancestry (reference)	Our cohort			
G: 95.00%	G: 95.37%	0.000; *p* = 1.000	*CYP3A4* (rs35599367)	0.187; *p* = 0.666
A: 5.00%	A: 4.63%		GG: 2,151 (91.0)	
Cunningham et al. (2022)			GA: 207 (8.8)	
			AA: 6 (0.2)	
A: 5.70%	A: 7.15%	0.082; *p* = 0.774	*CYP3A5* (rs776746)	0.985; *p* = 0.321
G: 94.30%	G: 92.85%		AA: 8 (0.4)	
Cunningham et al. (2022)			AG: 322 (13.6)	
			GG: 2,034 (86.0)	
T: 5.70%	T: 6.79%	0.082; *p* = 0.774	*CYP2J2* (rs890293)	1.778; *p* = 0.182
G: 94.30%	G: 93.21%		TT: 15 (0.6)	
Cunningham et al. (2022)			GT: 291 (12.3)	
			GG: 2,058 (87.1)	
C: 90.60%	C: 89.57%	0.058; *p* = 0.809	*ABCG2* (rs2231142)	1.922; *p* = 0.165
A: 9.40%	A: 10.43%		CC: 1,903 (80.5)	
Cunningham et al. (2022)			CA: 429 (18.1)	
			AA: 32 (1.4)	
T: 41.60%	T: 43.51%	0.082; *p* = 0.775	*ABCB1* (rs1128503)	0.777; *p* = 0.378
C: 58.40%	C: 56.49%		TT: 458 (19.4)	
Cunningham et al. (2022)			TC: 1,141 (48.3)	
			CC: 765 (32.3)	
T: 41.00%	T: 45.05%	0.202; *p* = 0.653	*ABCB1* (rs2032582)	0.465; *p* = 0.495
G: 57.30%	G: 54.95%		TT: 488 (20.6)	
Cunningham et al. (2022)			TG: 1,154 (48.8)	
			GG: 722 (30.6)	
T: 51.80%	T: 53.74%	0.080; *p* = 0.777	*ABCB1* (rs1045642)	0.262; *p* = 0.609
C: 48.20%	C: 46.26%		TT: 689 (29.1)	
Cunningham et al. (2022)			TC: 1,163 (49.2)	
			CC: 512 (21.7)	
G: 49.41%	G: 44.97%	0.321; *p* = 0.571	*ABCB1* (rs4148732)	1.770; *p* = 0.183
A: 50.59%	A: 55.03%		GG: 494 (20.9)	
Lähteenmäki et al. (2021)			GA: 1,138 (48.1)	
			AA: 732 (31.0)	

**TABLE 4:** Cox proportional hazards regression with time-varying analysis assessing the association of additive PK-related genetic variant models with DOACs bleeding risk.

*CYP3A4* (rs35599367)	HR (95%CI; *p*-value)
AA vs. AG vs. GG genotypes	
Model 1	0.876 (0.691–1.110); 0.274
Model 2	0.891 (0.708–1.122); 0.327
*CYP3A5* (rs776746)	HR (95%CI; *p*-value)
GG vs. GA vs. AA genotypes	
Model 1	0.960 (0.685–1.347); 0.814
Model 2	0.943 (0.687–1.294); 0.716
*CYP2J2* (rs890293)	HR (95%CI; *p*-value)
GG vs. GT vs. TT genotypes	
Model 1	1.133 (0.873–1.471); 0.349
Model 2	1.131 (0.871–1.468); 0.357
*ABCG2* (rs2231142)	HR (95%CI; *p*-value)
CC vs. CA vs. AA genotypes	
Model 1	1.076 (0.882–1.314); 0.469
Model 2	1.055 (0.863–1.289); 0.602
*ABCB1* (rs4148732)	HR (95%CI; *p*-value)
GG vs. GA vs. AA genotypes	
Model 1	1.113 (0.969–1.277); 0.129
Model 2	1.096 (0.956–1.256); 0.188
*ABCB1* C-G-C diplotypes	HR (95%CI; *p*-value)
Homozygous vs. hetero vs. other	
Model 1	0.999 (0.868–1.148); 0.983
Model 2	1.027 (0.895–1.179); 0.707

Model 1: Unadjusted model. Model 2: Fully adjusted for age, previous bleeding, Elixhauser comorbidities score, previous thromboembolism, smoking, normalized dose, and DOAC. The underlined genotypes were encoded as the risk genotype in the additive genetic model, and thus the HR for each variant was hypothesized to be > 1.

**TABLE 5:** Cox proportional hazards regression with time-varying analysis assessing the independent association of 1236C>T (rs1128503), 2677G>T/A (rs2032582), and 3435C>T (rs1045642) *ABCB1* haplotypes with bleeding from DOACs.

Haplotypes	Overall n (%)	Major + CRNM bleeding^a^		χ^2^; *p*-value^a^	HR (95%CI; *p*-value^a^	
		No	Yes		Unadjusted	Fully adjusted^b^
C-G-C	1,961 (41.5)	1,621 (65.7)	340 (63.1)	3.117; 0.210	0.999 (0.868–1.148); 0.983	1.027 (0.895–1.179); 0.707
T-T-T	1,888 (39.9)	1,546 (63.6)	342 (64.6)	2.012; 0.366	1.127 (0.976–1.301); 0.102	1.092 (0.948–1.258); 0.223
C-G-T	558 (11.8)	470 (22.7)	88 (20.1)	1.278; 0.528	0.826 (0.672–1.017); 0.071	0.794 (0.640–0.985); 0.036
T-T-C	90 (1.9)	71 (3.4)	19 (4.6)	2.772; 0.302	0.971 (0.641–1.471); 0.890	1.071 (0.699–1.642); 0.753
C-T-T	90 (1.9)	75 (3.8)	15 (3.6)	0.003; 0.958	0.959 (0.607–1.514); 0.856	0.970 (0.617–1.525); 0.893
T-G-C	74 (1.6)	65 (3.3)	9 (2.2)	1.118; 0.290	0.601 (0.292–1.236); 0.166	0.613 (0.290–1.298); 0.201
C-T-C	62 (1.3)	51 (2.6)	11 (2.7)	0.246; 0.884	0.896 (0.513–1.566); 0.700	0.970 (0.592–1.590); 0.905
T-G-T	5 (0.1)	5 (0.3)	0 (0.0)	0.192; 0.594	2.2542e^−06^ (8.035e^−07^–6.325e^−06^); <0.001	4.996e^−06^ (1.773e^−06^–1.408e^−05^); <0.001

^a^ CRNM: Clinically relavant non-major bleeding; χ^2^: chi-square; OR: odds ratio; 95%CI: 95% confidence interval.

^b^ Fully adjusted for age, previous bleeding, Elixhauser comorbidities score, previous thromboembolism, smoking, normalized dose, and DOAC.

## Discussion

To the best of our knowledge, this is the largest pharmacogenetic study to assess the association of PK-related genetic variants with the risk of bleeding in non-valvular AF white outpatients undergoing treatment with either rivaroxaban or apixaban. Eight SNVs known to be functionally involved with either the metabolism or excretion of DOACs were selected for analysis (Kuehl et al., 2001; Salama et al., 2006; Tomlinson et al., 2010; Wang et al., 2011). None of the eight SNVs met the pre-specified Bonferroni-corrected level of statistical significance for the risk of bleeding from DOACs. Our findings are in line with recent evidence from a candidate gene study that tested virtually the same genetic variants without any of them being associated with bleeding risk from apixaban (Attelind et al., 2022). In exploratory analyses, a tendency of association with a *p*-value <0.05 was observed between the *CYP3A5* rs776746 G carriers and reduced risk of bleeding from DOACs. However, the number of patients with the *CYP3A5* rs776746 AA genotype in this sample was extremally small (n = 8), and thus this result is most likely due to chance. Future pharmacogenetic studies should be conducted with this variant in AF patients of African ancestry, in whom the A allele is much more frequent (in more than 70%) (Cunningham et al., 2022). The *ABCB1* rs4148732 GG genotype also tended to be associated with an increased risk of bleeding on DOACs in exploratory analyses, and there was a large number of patients with the GG genotype (n = 494). However, this finding is also likely due to chance because it is not supported by previous pharmacogenetic studies of bleeding. None of the *ABCB1* rs4148732 genotypes was significantly associated with bleeding from apixaban in a previous study (Roşian et al., 2020a), and our results contradict another study finding that the *ABCB1* rs4148732GG was a *protective* genotype against bleeding from apixaban (Lähteenmäki et al., 2021). This previous study did not correct the level of significance for multiple comparisons (Bromley et al., 2009), and thus their previous finding for the *ABCB1* rs4148732GG genotype, like in our exploratory analysis, is most likely due to chance.

There are three main reasons that most likely explain the lack of significant association between the PK-related genetic variants and bleeding risk from DOACs in our findings. First, although genetic variants affecting the functionality of enzymes involved in the main metabolic pathways of rivaroxaban (CYP3A4 and CYP2J2) and apixaban (CYP3A4) have been tested, several other enzymes also play a role in the metabolism of these drugs (e.g., CYP1A2, CYP2C8, CYP2C9, CYP2C19, sulfotransferase). Therefore, these other enzymes may compensate for the genetic effects on the main pathway and preserve the overall metabolism of DOACs. These more complex PK pathways of rivaroxaban and apixaban differ from other well-known pharmacogenetic examples, in which the drug’s metabolism or transport is primarily dependent on a single enzyme or transporter (e.g., clopidogrel and *CYP2C19* or simvastatin and *SLCO1B1*) (Cooper-DeHoff et al., 2022; Lee et al., 2022). Furthermore, as opposed to a purely PK outcome, we assessed the clinical outcome of bleeding, and PK is just one side of this pharmacological double-edged sword. The coagulation cascade is highly complex and works from the interaction of multiple factors and cofactors, and genetic variants affecting pharmacodynamic (PD) changes of DOACs were not assessed in this or any other pharmacogenetic study to date.

Second, the effects of these genetic variants on DOAC exposure may not be strong enough to translate into differences in clinical outcomes. Bleeding complications during DOAC treatment have been shown to be more frequent when peak DOAC plasma levels reached 266% for rivaroxaban and 156% for apixaban compared to baseline (Testa et al., 2019). However, the only two pharmacogenetic studies with significant results for peak plasma levels of DOACs showed smaller effect sizes. Increases of 106% and 26% for peak plasma concentrations of rivaroxaban and apixaban were respectively reported in individuals with the *ABCB1* rs4148732 GG (Cosmi et al., 2019) and AA genotypes compared to the homozygous counterparts (Dimatteo et al., 2016). The notion that genetic variants would have small effects on DOAC exposure that would not translate into clinical outcomes is supported by recent evidence that carriers of T allele *ABCG2* (rs2231142) have a 5% and 17% higher exposure to apixaban in heterozygous and homozygous, respectively, without any association to bleeding risk from this DOAC (Attelind et al., 2022). Of course, future studies need to confirm these findings, but this preliminary evidence suggests that these genetic variants would not have an effect size large enough on the PK to translate into clinical hemorrhagic outcomes.

The third reason that could explain the lack of significant association in our study is the candidate gene study design. Candidate gene studies are hypothesis-driven with the advantage of having high statistical power in detecting gene-drug associations. However, these studies miss the thousands of other genes and millions of other variants present in the genome–such as the PD-related genetic variants mentioned above or other yet unknown PK variants. Indeed, recent evidence has shown that the vast majority of candidate variants selected for pharmacogenetic studies (98%) are not the best candidates (Linskey et al., 2021). Hence, genome-wide association studies (GWAS) are crucial to discover the most important genetic variants underlying bleeding from DOACs. Although recent evidence from a large GWAS has shown that the genetic variant in the drug transporter gene *ABCG2* is associated with the pharmacokinetics of apixaban, this genetic variant had a small influence on drug exposure (Attelind et al., 2022). Future GWAS continues to be needed to identify and uncover the genetic mechanisms underlying the risk of bleeding from DOACs. Another weakness of candidate gene studies lies in the fact that its design also misses polygenic effects (i.e., the effects of multiple genetic variants combined). The most recent evidence shows that interindividual variability in drug response is explained by polygenic inheritance (Lanfear et al., 2020; Johnson et al., 2022). Therefore, it may be that each of the genetic variants has a small effect size on bleeding that is not statistically significant if analyzed individually through a candidate gene study, but perhaps the small effect sizes need to be aggregated into a polygenic score weighted so that its clinical significance can then be determined.

Although our study has some major strengths, such as having clinical outcomes rather than blood levels of DOACs and testing the largest patient sample to date, some limitations must be taken into consideration. This was an observational study, and our findings are limited to a single health system that primarily serves southeastern Michigan. All data were obtained from the EHR, which limits the research to certain variables. Although our data collection has followed standardized protocols, there is no standardization for the clinical care routine and, as a limitation of observational studies, unmeasured residual confounding may have been incorporated. The drug prescribing was defined from EHR data, and clinician reasoning could not be obtained to determine the thought process of prescribing these drugs. We did not have access to pharmacy prescription fill records, so adherence to the DOAC therapy could not be assessed. Finally, not all potential candidates (e.g., rs1042028 in *SULT1A1*) were covered by the genotyping performed by Michigan Genomics Initiative.

## Conclusion

In conclusion, eight functional PK-related genetic variants were not significantly associated with the bleeding risk in more than 2,000 white Americans diagnosed with non-valvular atrial fibrillation and treated with either rivaroxaban or apixaban in a large health system.

## Data Availability

The datasets presented in this article are not readily available because of patient privacy and confidentiality restrictions. Requests to access the datasets should be directed to https://precisionhealth.umich.edu/tools-resources/data-access-tools/how-to-access-data-tools-analytic-environments/
