# Supplementary material for: Eight pharmacokinetic genetic variants are not associated with the risk of bleeding from direct oral anticoagulants in non-valvular atrial fibrillation patients
Source: Front Pharmacol. 2022 Nov 24;13:1007113. doi: 10.3389/fphar.2022.1007113 (PMC9730333; doi:10.3389/fphar.2022.1007113)
Supplement: Supplementary file 1 [file DataSheet1.docx]

Supplementary Material

**Supplementary table 1.** Summary of evidence from previous pharmacogenetic studies on rivaroxaban and apixaban.

| References | Study design, sample size, endpoints & DOAC | Genetic variant(s) | Summary of PK and/or clinical results |
| --- | --- | --- | --- |
| (Dimatteo et al., 2016)  PMID: 27434880 | - 80 non-valvular AF patients on apixaban - Apixaban peak and trough concentrations | - *ABCB1* c.2482-2236G>A (rs4148738, intron 18) | AA genotype had higher peak concentration of apixaban (263.7 ng/dL) than carriers of the G allele (209.2 ng/dL, p = 0.048). |
| (Lorenzini et al., 2016)  PMID: 28066243 | - Case report of a rivaroxaban-induced hemorrhage in an AF patient | - *ABCB1* c.2677G>T (p.Ala893Ser, rs2032582) - *ABCB1* c.3435C>T (p.Ile1145=, rs1045642) | *ABCB1* c.2677G>T (p.Ala893Ser, rs2032582): TT genotype  *ABCB1* c.3435C>T (p.Ile1145=, rs1045642): TT genotype |
| (Gouin-Thibault et al., 2017)  PMID: 27893182 | - 60 healthy male volunteers on a single dose of rivaroxaban associated or not with clarithromycin - Peak plasma concentration and area under the curve (AUC) of rivaroxaban | - *ABCB1* c.2677G>T (p.Ala893Ser, rs2032582) - *ABCB1* c.3435C>T (p.Ile1145=, rs1045642) | *ABCB1* 2677-3435 haplotype was not significantly associated with peak plasma concentration and AUC of rivaroxaban. |
| (Ueshima et al., 2017)  PMID: 28678049 | - 44 AF patients on apixaban - Plasma trough concentration/dose (C/D) ratio of apixaban | - *ABCB1* c.1236C>T (p.Gly412=, rs1128503) - *ABCB1* c.2677G>T (p.Ala893Ser, rs2032582) - *ABCB1* c.3435C>T (p.Ile1145=, rs1045642) - *ABCG2* c.421C>A (p.Gln141Lys, rs2231142) - *CYP3A5* g.6981A>G (splice defect, CYP3A5*3, rs776746) | *ABCB1* (rs1128503 and rs1045642): no significant differences in plasma C/D ratio of apixaban.  *ABCB1* (rs2032582): plasma C/D ratio of apixaban was slightly higher in GG genotype than heterozygous (p <0.05).  *ABCG2* (rs2231142): plasma trough C/D ratio of apixaban was significantly higher in AA than CC genotype (p <0.01).  *CYP3A5* (rs776746): plasma trough C/D ratio of apixaban was higher in the G carriers than AA genotype (p <0.05). |
| (Kryukov et al., 2018)  PMID: 29606886 | - 17 AF patients with cardioembolic stroke on apixaban - Apixaban plasma concentrations | - *ABCB1* c.3435C>T (p.Ile1145=, rs1045642) - *ABCB1* c.2482-2236G>A (rs4148738, intron 18) - *CYP3A5* g.6981A>G (splice defect, CYP3A5*3, rs776746) | None of the genetic variants were significantly related to the pharmacokinetic parameters of apixaban. |
| (Sennesael et al., 2018)  PMID: 30455596 | - Case series including 10 rivaroxaban-treated patients admitted for bleeding, of which 3 of them were genotyped. | - *ABCB1* c.1236C>T (p.Gly412=, rs1128503) - *ABCB1* c.2677G>T (p.Ala893Ser, rs2032582) - *ABCB1* c.3435C>T (p.Ile1145=, rs1045642) - *ABCB1* c.2482-2236G>A (rs4148738, intron 18) | Three genotyped patients showed higher-than-expected rivaroxaban levels:  *ABCB1* (rs1128503): all 3 patients having CT genotype  *ABCB1* (rs2032582): all 3 patients having GT genotype  *ABCB1* (rs1045642): 2 patients having CT and 1 having TT genotype  *ABCB1* (rs4148738): all 3 patients having GA genotype |
| (Sychev et al., 2018)  DOI: 10.24075/vrgmu.2018.068 | - 65 patients undergoing total hip or knee replacement surgery on rivaroxaban - Peak and trough steady-state rivaroxaban concentrations | - *ABCB1* c.3435C>T (p.Ile1145=, rs1045642) - *CYP3A5* g.6981A>G (splice defect, CYP3A5*3, rs776746) | *ABCB1* (rs1045642): ΔPT ≤0 was significantly more frequent among carriers of the C allele than the genotype TT (p = 0.027).  *CYP3A5* (rs776746): there is no significant difference in ΔPT across genotypes. |
| (Ueshima et al., 2018)  PMID: 29457840 | - 81 AF patients on apixaban - Pharmacokinetic parameters of apixaban (apparent oral clearance and apparent volume of distribution) | - *ABCB1* c.1236C>T (p.Gly412=, rs1128503) - *ABCB1* c.2677G>T (p.Ala893Ser, rs2032582) - *ABCB1* c.3435C>T (p.Ile1145=, rs1045642) - *ABCG2* c.421C>A (p.Gln141Lys, rs2231142) - *CYP3A5* g.6981A>G (splice defect, CYP3A5*3, rs776746) | *CYP3A5* (rs776746): AA genotype had a significant higher trough concentration of apixaban than the G allele carriers (p <0.001).  *ABCG2* (rs2231142): C allele carriers had a significant higher trough concentration of apixaban than AA genotype (p <0.001). |
| (Cosmi et al., 2019)  DOI: https://doi.org/10.12970/2311-052X.2019.07.02 | - 142 genotyped patients with either VTE or AF on rivaroxaban - Anti-Xa activity for apixaban and rivaroxaban on plasmas at trough and at peak | - *ABCB1* c.2482-2236G>A (rs4148738, intron 18) | GG genotype had a significantly higher peak (289 ng/mL), but not trough, of rivaroxaban levels than A carriers (244 and 140 ng/mL, p = 0.041). No difference was observed for apixaban. |
| (Huppertz et al., 2019)  PMID: 31464657 | - Case report of an AF patient on apixaban admitted due to suspected acute stroke | - *ABCB1* c.2677G>T (p.Ala893Ser, rs2032582) - *ABCB1* c.3435C>T (p.Ile1145=, rs1045642) - *ABCG2* c.421C>A (p.Gln141Lys, rs2231142) - *CYP3A5* g.6981A>G (splice defect, CYP3A5*3, rs776746) | High-than-expected blood levels of apixaban with a moderate renal dysfunction  *ABCB1* (rs2032582): TT genotype  *ABCB1* (rs1045642): TT genotype  *ABCG2* (rs2231142): CA genotype  *CYP3A5* (rs776746): GG genotype |
| (Sychev et al., 2019)  PMID: 31617197 | - 78 patients undergoing total hip or knee replacement surgery on rivaroxaban | - *ABCB1* c.3435C>T (p.Ile1145=, rs1045642) - *ABCB1* c.2482-2236G>A (rs4148738, intron 18) - *CYP3A4* c.15389C>T (rs35599367, intron 6) - *CYP3A5* g.6981A>G (splice defect, CYP3A5*3, rs776746) | None of the SNVs showed a significant difference in the steady-state peak concentration of rivaroxaban. |
| (Gulilat et al., 2020)  PMID: 31564018 | - 358 AF patients on apixaban | - *ABCG2* c.34G>A (p.Val12Met, rs2231137) - *ABCG2* c.421C>A (p.Gln141Lys, rs2231142) - *ABCB1* c.3435C>T (p.Ile1145=, rs1045642) - *CYP3A4* c.15389C>T (rs35599367, intron 6) - *CYP3A5* g.6981A>G (splice defect, CYP3A5*3, rs776746) | *ABCG2* (rs2231142): carriers of the A allele were predicted to have increased peak and trough concentrations than CC genotype (p = 0.040). |
| (Roşian et al., 2020a)  PMID: 32961964 | - 114 AF patients on apixaban - Non-major bleeding according to the ISTH criteria | - *ABCB1* c.3435C>T (p.Ile1145=, rs1045642) - *ABCB1* c.2482-2236G>A (rs4148738, intron 18) | None of the genetic variants were significantly associated with non-major bleeding risk |
| (Roşian et al., 2020b)  PMID: 32316515 | - 53 non-valvular AF patients on apixaban - Trough and peak plasma concentrations of apixaban | - *ABCB1* c.3435C>T (p.Ile1145=, rs1045642) - *ABCB1* c.2482-2236G>A (rs4148738, intron 18) | None of the genetic variants were significantly related to the pharmacokinetic parameters of apixaban |
| (Sychev et al., 2020)  PMID: 32158254 | - 103 patients with non-valvular AF on rivaroxaban | - *ABCB1* c.3435C>T (p.Ile1145=, rs1045642) - *ABCB1* c.2482-2236G>A (rs4148738, intron 18) - *CYP2C19* c.681G>A (p.Pro227=, rs4244285) - *CYP2C19* c.-806C>T (rs12248560) - *CYP3A5* g.6981A>G (splice defect, CYP3A5*3, rs776746) | None of the SNVs showed a significant difference in the blood concentration of rivaroxaban |
| (Lähteenmäki et al., 2021)  PMID: 34043814 | - Retrospective biobank analysis of 1,806 Finnish individuals on apixaban or rivaroxaban - Bleeding or thromboembolic events defined by diagnosis codes | - *ABCG2* c.421C>A (p.Gln141Lys, rs2231142) - *ABCB1* c.3435C>T (p.Ile1145=, rs1045642) - *ABCB1* c.2677G>T (p.Ala893Ser, rs2032582) - *ABCB1* c.2677G>A (p.Ala893Thr, rs2032582) - *ABCB1* c.2482-2236G>A (rs4148738, intron 18) - *ABCB1* c.1236C>T (p.Gly412=, rs1128503) - *CYP3A5* g.6981A>G (splice defect, CYP3A5*3, rs776746) | The *ABCB1* c.2482-2236G>A (rs4148738) SNV was associated with a lower risk for bleeding events [0.37 (0.16-0.89), p = 0.025] in apixaban users. |

DOAC: direct oral anticoagulant; PK: pharmacokinetics; SNV: single nucleotide variant; AF: atrial fibrillation; PT: prothrombin time; ISTH: International Society of Thrombosis and Haemostasis.

**Supplementary Table 2.** Baseline characteristics of patients stratified by major bleeding.

| **Characteristics** | **Overall** | **Major bleeding** | | **p-value** |
| --- | --- | --- | --- | --- |
|  |  | **No** | **Yes** |  |
| **Self-identified white participants, n (%)** | 2364 (100.0) | 2267 (95.9) | 97 (4.1) | __ |
| **Age, years** | 68.3 (13.6) | 68.1 (13.5) | 74.1 (9.1) | **<0.001** |
| **Sex, n (%)**  Female  Male | 758 (32.1)  1606 (67.9) | 720 (31.8)  1547 (68.2) | 38 (39.2)  59 (60.8) | 0.125 |
| **Ethnicity, n (%)**  Non-Hispanic or Latino  Hispanic or Latino  Unknown | 2312 (97.8)  10 (0.4)  42 (1.8) | 2216 (97.8)  10 (0.4)  41 (1.8) | 96 (99.0)  0 (0.0)  1 (1.0) | 0.684 |
| **DOAC, n (%)**  Rivaroxaban  Apixaban  Both DOACs not simultaneously | 802 (33.9)  1324 (56.0)  238 (10.1) | 772 (34.1)  1276 (56.3)  219 (9.7) | 30 (30.9)  48 (49.5)  19 (19.6) | **0.006** |
| **Daily dose of DOAC, mg**  Rivaroxaban  Apixaban | 19.3 ± 3.5  7.8 ± 4.1 | 19.3 ± 3.5  7.7 ± 4.1 | 18.7 ± 2.7  8.7 ± 3.6 | 0.308  0.056 |
| **Cumulative dose of DOAC, g**  Rivaroxaban  Apixaban | 16.5 ± 15.5  7.2 ± 6.5 | 16.6 ± 15.5  7.2 ± 6.5 | 16.0 ± 14.7  7.1 ± 6.3 | 0.816  0.424 |
| **Drug-drug interactions with DOACs, n (%)**  CYP/p-gp inhibitors  CYP/p-gp inducers | 1046 (44.5)  33 (1.4) | 983 (43.4)  31 (1.4) | 63 (65.0)  2 (2.1) | **<0.001**  0.568 |
| **Previous surgery or trauma, n (%)** | 1449 (61.3) | 1381 (60.9) | 68 (70.1) | 0.069 |
| **Transcatheter aortic valve replacement, n (%)** | 22 (0.9) | 17 (0.8) | 5 (5.2) | **<0.001** |
| **Previous bleeding, n (%)** | 491 (20.8) | 465 (20.5) | 26 (26.8) | 0.135 |
| **Previous stroke, n (%)** | 50 (2.1) | 47 (2.1) | 3 (3.1) | 0.494 |
| **Previous transient ischemic attack, n (%)** | 37 (1.6) | 35 (1.5) | 2 (2.1) | 0.687 |
| **Previous thromboembolism, n (%)** | 90 (3.8) | 85 (3.8) | 5 (5.2) | 0.479 |
| **CHA_2_DS_2_-VASc score** | 2.4 ± 1.5 | 2.4 ± 1.5 | 3.0 ± 1.7 | **<0.001** |
| **Elixhauser comorbidities score** | 11.0 ± 11.3 | 10.9 ± 11.2 | 13.9 ± 12.0 | **<0.001** |
| **HAS-BLED score** | 1.3 ± 1.1 | 1.3 ± 1.0 | 1.6 ± 1.1 | **0.020** |
| **ATRIA score** | 2.3 ± 2.0 | 2.3 ± 2.0 | 3.4 ± 1.9 | **<0.001** |
| **Smoking habit, n (%)**  Never  Current  Former  Unknown | 1052 (44.5)  67 (2.8)  1222 (51.7)  23 (1.0) | 1022 (45.1)  67 (3.0)  1155 (51.0)  23 (1.0) | 30 (30.9)  0 (0.0)  67 (69.1)  0 (0.0) | **0.003** |
| **Body mass index, kg/m^2^** | 29.9 (8.4) | 29.9 (8.4) | 29.5 (8.2) | 0.618 |
| **Systolic blood pressure, mmHg** | 127.5 (21.5) | 127.5 (22.0) | 127.5 (17.5) | 0.905 |
| **Diastolic blood pressure, mmHg** | 71.0 (11.5) | 71.0 (11.5) | 67.5 (11.0) | **<0.001** |
| **Creatinine clearance, mL/min** | 92.2 (49.0) | 92.6 (49.2) | 75.5 (42.5) | **<0.001** |
| **Platelet count, 10^9^/L** | 209.8 (74.0) | 209.5 (73.0) | 216.0 (95.0) | 0.193 |
| **Hemoglobin, g/dL** | 13.5 (2.5) | 13.5 (2.4) | 12.4 (3.4) | **<0.001** |
| **Follow-up time, (days)** | 828.2 ± 739.8 | 830.0 ± 740.0 | 787.8 ± 735.6 | 0.195 |

DOAC: Direct Oral Anticoagulants; CYP: Cytochrome P450; p-gp: p-glycoprotein.

**Supplementary Table 3.** Baseline characteristics of patients stratified by DOAC treatment.

| **Characteristics** | **Overall** | **DOAC** | | **p-value** |
| --- | --- | --- | --- | --- |
|  |  | **Apixaban** | **Rivaroxaban** |  |
| **Self-identified white participants, n (%)** | 2,364 (100.0) | 1,324 (56.0) | 1,040 (44.0) | __ |
| **Age, years** | 68.3 (13.6) | 69.1 (13.6) | 67.5 (13.2) | **0.001** |
| **Sex, n (%)**  Female  Male | 758 (32.1)  1,606 (67.9) | 422 (31.9)  902 (68.1) | 336 (32.3)  704 (67.7) | 0.822 |
| **Ethnicity, n (%)**  Non-Hispanic or Latino  Hispanic or Latino  Unknown | 2,312 (97.8)  10 (0.4)  42 (1.8) | 1,301 (98.3)  2 (0.1)  21 (1.6) | 1,011 (97.2)  8 (0.8)  21 (2.0) | 0.051 |
| **Drug-drug interactions with DOACs, n (%)**  CYP/p-gp inhibitors  CYP/p-gp inducers | 1,046 (44.5)  33 (1.4) | 552 (41.7)  15 (1.1) | 494 (47.5)  18 (1.7) | **0.005**  0.219 |
| **Previous surgery or trauma, n (%)** | 1,449 (61.3) | 858 (64.8) | 591 (56.8) | **<0.001** |
| **Transcatheter aortic valve replacement, n (%)** | 22 (0.9) | 20 (1.5) | 2 (0.2) | **0.001** |
| **Composite endpoint, n (%)** | 412 (17.4) | 200 (15.1) | 212 (20.4) | **0.001** |
| **Previous bleeding, n (%)** | 491 (20.8) | 328 (24.8) | 163 (15.7) | **<0.001** |
| **Previous stroke, n (%)** | 50 (2.1) | 38 (2.9) | 12 (1.2) | **0.004** |
| **Previous transient ischemic attack, n (%)** | 37 (1.6) | 23 (1.7) | 14 (1.4) | 0.447 |
| **Previous thromboembolism, n (%)** | 90 (3.8) | 49 (3.7) | 41 (3.9) | 0.761 |
| **CHA_2_DS_2_-VASc score** | 2.4 ± 1.5 | 2.6 ± 1.6 | 2.2 ± 1.4 | **<0.001** |
| **Elixhauser comorbidities score** | 11.0 ± 11.3 | 12.5 ± 11.7 | 9.0 ± 10.4 | **<0.001** |
| **HAS-BLED score** | 1.3 ± 1.1 | 1.4 ± 1.1 | 1.1 ± 0.9 | **<0.001** |
| **ATRIA score** | 2.3 ± 2.0 | 2.5 ± 2.1 | 2.0 ± 1.9 | **<0.001** |
| **Smoking habit, n (%)**  Never  Current  Former  Unknown | 1,052 (44.5)  67 (2.8)  1,222 (51.7)  23 (1.0) | 602 (45.5)  40 (3.0)  668 (50.4)  14 (1.1) | 450 (43.3)  27 (2.6)  554 (53.2)  9 (0.9) | 0.548 |
| **Body mass index, kg/m^2^** | 29.9 (8.4) | 29.5 (8.2) | 30.4 (8.7) | **0.013** |
| **Systolic blood pressure, mmHg** | 127.5 (21.5) | 128.0 (21.5) | 127.0 (22.0) | 0.652 |
| **Diastolic blood pressure, mmHg** | 71.0 (11.5) | 71.0 (11.8) | 71.0 (11.0) | 0.159 |
| **Creatinine clearance, mL/min** | 92.2 (49.0) | 88.7 (47.1) | 95.7 (51.0) | **<0.001** |
| **Platelet count, 10^9^/L** | 209.8 (74.0) | 210.0 (72.0) | 209.0 (75.3) | 0.835 |
| **Hemoglobin, g/dL** | 13.5 (2.5) | 13.4 (2.5) | 13.5 (2.4) | 0.345 |
| **Follow-up time, (days)** | 828.2 ± 739.8 | 732.8 ± 648.1 | 949.8 ± 826.6 | **<0.001** |

DOAC: Direct Oral Anticoagulants; CYP: Cytochrome P450; p-gp: p-glycoprotein.

**Supplementary Table 4.** Cox proportional hazards regression with time-varying analysis assessing the association of dominant and recessive PK-related genetic variant models with DOACs bleeding risk.

| ***CYP3A4* (rs35599367)** | **HR (95%CI; p-value)** |
| --- | --- |
| **AA genotype *vs*. G carriers**  Model 1  Model 2 | 0.937 (0.114-7.688); 0.952  1.053 (0.150-7.378); 0.959 |
| **A carriers *vs*. GG genotype**  Model 1  Model 2 | 0.786 (0.556-1.111); 0.173  0.798 (0.568-1.123); 0.196 |
| ***CYP3A5* (rs776746)** | **HR (95%CI; p-value)** |
| **GG genotype *vs*. A carriers**  Model 1  Model 2 | 1.022 (0.756-1.383); 0.886  1.005 (0.754-1.339); 0.974 |
| **G carriers *vs*. AA genotype**  Model 1  Model 2 | 0.256 (0.059-1.114); 0.069  0.233 (0.093-0.582); **0.002** |
| ***CYP2J2* (rs890293)** | **HR (95%CI; p-value)** |
| **GG genotype *vs*. T carriers**  Model 1  Model 2 | 1.123 (0.848-1.486); 0.418  1.103 (0.835-1.458); 0.489 |
| **G carriers *vs*. TT genotype**  Model 1  Model 2 | 1.587 (0.549-4.588); 0.394  2.065 (0.811-5.256); 0.128 |
| ***ABCG2* (rs2231142)** | **HR (95%CI; p-value)** |
| **CC genotype *vs*. A carriers**  Model 1  Model 2 | 1.143 (0.902-1.448); 0.268  1.128 (0.891-1.429); 0.317 |
| **C carriers *vs*. AA genotype**  Model 1  Model 2 | 0.736 (0.327-1.655); 0.458  0.665 (0.292-1.515); 0.331 |
| ***ABCB1* (rs4148732)** | **HR (95%CI; p-value)** |
| **G carriers *vs*. AA genotype**  Model 1  Model 2 | 1.049 (0.852-1.292); 0.649  1.042 (0.844-1.286); 0.700 |
| **GG genotype *vs*. A carriers**  Model 1  Model 2 | 1.293 (1.035-1.615); **0.024**  1.249 (1.001-1.557); **0.049** |
| ***ABCB1* C-G-C diplotypes** | **HR (95%CI; p-value)** |
| **Homozygous *vs.* hetero *vs.* other**  Model 1  Model 2 | 0.999 (0.868-1.148); 0.983  1.027 (0.895-1.179); 0.707 |

**Model 1:** Unadjusted model. **Model 2:** Fully adjusted for age, previous bleeding, Elixhauser comorbidities score, previous thromboembolism, smoking, normalized dose, and DOAC.

The underlined genotypes were encoded as the risk genotype in the dominant and recessive genetic models.

**Supplementary Table 5.** Cox proportional hazards regression with time-varying analysis assessing the association of PK-related genetic variants with the risk of bleeding from rivaroxaban and apixaban.

| ***CYP3A4* (rs35599367)** | **Rivaroxaban**  **HR (95%CI; p-value)** | **Apixaban**  **HR (95%CI; p-value)** |
| --- | --- | --- |
| **AA *vs*. AG *vs*. GG genotypes**  Model 1  Model 2 | 0.876 (0.691-1.110); 0.274  0.896 (0.711-1.130); 0.355 | 0.872 (0.556-1.369); 0.552  0.785 (0.499-1.234); 0.294 |
| **AA genotype *vs*. G carriers**  Model 1  Model 2 | 0.937 (0.114-7.688); 0.952  1.075 (0.163-7.103); 0.940 | 2.474 (0.423-14.450); 0.315  2.559 (0.579-11.319); 0.215 |
| **A carriers *vs*. GG genotype**  Model 1  Model 2 | 0.786 (0.556-1.111); 0.173  0.785 (0.557-1.106); 0.166 | 0.841 (0.533-1.327); 0.457  0.754 (0.478-1.187); 0.222 |
| ***CYP3A5* (rs776746)** | **HR (95%CI; p-value)** | **HR (95%CI; p-value)** |
| **GG *vs*. GA *vs*. AA genotypes**  Model 1  Model 2 | 0.924 (0.613-1.390); 0.703  0.966 (0.649-1.437); 0.865 | 1.056 (0.628-1.776); 0.838  1.156 (0.730-1.830); 0.536 |
| **GG genotype *vs*. A carriers**  Model 1  Model 2 | 0.976 (0.643-1.481); 0.908  1.021 (0.682-1.529); 0.921 | 0.955 (0.623-1464); 0.832  1.061 (0.705-1.597); 0.777 |
| **G carriers *vs*. AA genotype**  Model 1  Model 2 | 0.164 (0.069-0.389); **<0.001**  0.189 (0.070-0.509); **<0.001** | 4.557 (0.689-30.140); 0.116  4.107 (1.364-12.364); **0.012** |
| ***CYP2J2* (rs890293)** | **HR (95%CI; p-value)** | **HR (95%CI; p-value)** |
| **GG *vs*. GT *vs*. TT genotypes**  Model 1  Model 2 | 1.145 (0.801-1.635); 0.457  1.139 (0.801-1.619); 0.470 | 1.124 (0.776-1.628); 0.536  1.139 (0.779-1.666); 0.501 |
| **GG genotype *vs*. T carriers**  Model 1  Model 2 | 1.149 (0.766-1.722); 0.502  1.124 (0.754-1.676); 0.566 | 1.111 (0.759-1.626); 0.590  1.113 (0.759-1.634); 0.583 |
| **G carriers *vs*. TT genotype**  Model 1  Model 2 | 1.385 (0.414-4.628); 0.597  1.599 (0.543-4.711); 0.394 | 2.068 (0.342-12.520); 0.429  3.906 (0.990-15.411); 0.052 |
| ***ABCG2* (rs2231142)** | **HR (95%CI; p-value)** | **HR (95%CI; p-value)** |
| **CC *vs*. CA *vs*. AA genotypes**  Model 1  Model 2 | 1.058 (0.801-1.397); 0.693  1.030 (0.765-1.388); 0.844 | 1.048 (0.794-1.383); 0.742  1.054 (0.811-1.369); 0.694 |
| **CC genotype *vs*. A carriers**  Model 1  Model 2 | 1.173 (0.818-1.682); 0.385  1.158 (0.807-1.661); 0.426 | 1.062 (0.783-1.441); 0.700  1.062 (0.783-1.440); 0.699 |
| **C carriers *vs*. AA genotype**  Model 1  Model 2 | 0.538 (0.200-1.448); 0.220  0.468 (0.140-1.564); 0.217 | 0.994 (0.321-3.075); 0.991  1.063 (0.473-2.388); 0.882 |
| ***ABCB1* (rs4148732)** | **HR (95%CI; p-value)** | **HR (95%CI; p-value)** |
| **GG *vs*. GA *vs*. AA genotypes**  Model 1  Model 2 | 1.158 (0.948-1.414); 0.152  1.121 (0.916-1.372); 0.268 | 1.069 (0.889-1.286); 0.477  1.068 (0.893-1.277); 0.474 |
| **G carriers *vs*. AA genotype**  Model 1  Model 2 | 1.036 (0.762-1.410); 0.821  1.010 (0.739-1.381); 0.950 | 1.043 (0.797-1.366); 0.759  1.075 (0.817-1.415); 0.607 |
| **GG genotype *vs*. A carriers**  Model 1  Model 2 | 1.475 (1.096-1.985); **0.010**  1.391 (1.019-1.900); **0.038** | 1.156 (0.841-1.588); 0.372  1.116 (0.819-1.519); 0.487 |
| ***ABCB1* diplotypes** | **HR (95%CI; p-value)** | **HR (95%CI; p-value)** |
| **Homozygous *vs.* hetero *vs.* other**  Model 1  Model 2 | 0.978 (0.795-1.203); 0.834  1.027 (0.836-1.260); 0.803 | 1.002 (0.833-1.205); 0.983  1.036 (0.863-1.244); 0.702 |

**Model 1:** Unadjusted model. **Model 2:** Fully adjusted for age, previous bleeding, Elixhauser comorbidities score, previous thromboembolism, smoking, normalized dose, and DOAC.

The underlined genotypes were encoded as the risk genotype in the additive, dominant and recessive genetic models.

**REFERENCES**

Cosmi, B., Salomone, L., Cini, M., Guazzaloca, G., and Legnani, C. (2019). Observational Study of the Inter-Individual Variability of the Plasma Concentrations of Direct Oral Anticoagulants (Dabigatran, Rivaroxaban, Apixaban) and the Effect of rs4148738 Polymorphism of ABCB1. *Journal of Cardiology and Therapeutics* 7, 8–14. doi: 10.12970/2311-052x.2019.07.02.

Dimatteo, C., D’Andrea, G., Vecchione, G., Paoletti, O., Tiscia, G. L., Santacroce, R., et al. (2016). ABCB1 SNP rs4148738 modulation of apixaban interindividual variability. *Thrombosis Research* 145, 24–26. doi: 10.1016/j.thromres.2016.07.005.

Gouin-Thibault, I., Delavenne, X., Blanchard, A., Siguret, V., Salem, J. E., Narjoz, C., et al. (2017). Interindividual variability in dabigatran and rivaroxaban exposure: contribution of ABCB1 genetic polymorphisms and interaction with clarithromycin. *Journal of Thrombosis and Haemostasis*. doi: 10.1111/jth.13577.

Gulilat, M., Keller, D., Linton, B., Pananos, A. D., Lizotte, D., Dresser, G. K., et al. (2020). Drug interactions and pharmacogenetic factors contribute to variation in apixaban concentration in atrial fibrillation patients in routine care. *Journal of Thrombosis and Thrombolysis* 49, 294–303. doi: 10.1007/s11239-019-01962-2.

Huppertz, A., Grond-Ginsbach, C., Dumschat, C., Foerster, K. I., Burhenne, J., Weiss, J., et al. (2019). Unexpected excessive apixaban exposure: case report of a patient with polymorphisms of multiple apixaban elimination pathways. *BMC Pharmacol Toxicol* 20, 53. doi: 10.1186/s40360-019-0331-9.

Kryukov, A. V., Sychev, D. A., Andreev, D. A., Ryzhikova, K. A., Grishina, E. A., Ryabova, A. V., et al. (2018). Influence of ABCB1 and CYP3A5 gene polymorphisms on pharmacokinetics of apixaban in patients with atrial fibrillation and acute stroke. *Pharmacogenomics and Personalized Medicine* 11, 43–49. doi: 10.2147/PGPM.S157111.

Lähteenmäki, J., Vuorinen, A.-L., Pajula, J., Harno, K., Lehto, M., Niemi, M., et al. (2021). Pharmacogenetics of Bleeding and Thromboembolic Events in Direct Oral Anticoagulant Users. *Clinical Pharmacology & Therapeutics* 110, 768–776. doi: 10.1002/cpt.2316.

Lorenzini, K. I., Daali, Y., Fontana, P., Desmeules, J., and Samer, C. (2016). Rivaroxaban-induced hemorrhage associated with ABCB1 genetic defect. *Frontiers in Pharmacology*. doi: 10.3389/fphar.2016.00494.

Roşian, A. N., Iancu, M., Trifa, A. P., Roşian, Ş. H., Mada, C., Gocan, C. P., et al. (2020a). An exploratory association analysis of ABCB1 rs1045642 and ABCB1 rs4148738 with non-major bleeding risk in atrial fibrillation patients treated with dabigatran or apixaban. *Journal of Personalized Medicine* 10, 1–14. doi: 10.3390/jpm10030133.

Roşian, A. N., Roşian, Ş. H., Kiss, B., Ştefan, M. G., Trifa, A. P., Ober, C. D., et al. (2020b). Interindividual variability of apixaban plasma concentrations: Influence of clinical and genetic factors in a real-life cohort of atrial fibrillation patients. *Genes* 11. doi: 10.3390/genes11040438.

Sennesael, A. L., Larock, A. S., Douxfils, J., Elens, L., Stillemans, G., Wiesen, M., et al. (2018). Rivaroxaban plasma levels in patients admitted for bleeding events: Insights from a prospective study. *Thrombosis Journal*. doi: 10.1186/s12959-018-0183-3.

Sychev, D. A., Baturina, O. A., Mirzaev, K. B., Rytkin, E., Ivashchenko, D. V., Andreev, D. A., et al. (2020). Cyp2c19*17 may increase the risk of death among patients with an acute coronary syndrome and non-valvular atrial fibrillation who receive clopidogrel and rivaroxaban. *Pharmacogenomics and Personalized Medicine* 13, 29–37. doi: 10.2147/PGPM.S234910.

Sychev, D. A., Minnigulov, R. M., Ryzhikova, K. A., Yudina, I. Y., Lychagin, A. V., and Morozova, T. E. (2018). Evaluation of the rivaroxaban-influenced effect of ABCB1 and CYP3A5 gene polymorphisms on prothrombin time in patients after total hip or knee replacement surgery. *Bulletin of Russian State Medical University*. doi: 10.24075/brsmu.2018.068.

Sychev, D., Minnigulov, R., Bochkov, P., Ryzhikova, K., Yudina, I., Lychagin, A., et al. (2019). Effect of CYP3A4, CYP3A5, ABCB1 Gene Polymorphisms on Rivaroxaban Pharmacokinetics in Patients Undergoing Total Hip and Knee Replacement Surgery. *High Blood Pressure and Cardiovascular Prevention* 26, 413–420. doi: 10.1007/s40292-019-00342-4.

Ueshima, S., Hira, D., Fujii, R., Kimura, Y., Tomitsuka, C., Yamane, T., et al. (2017). Impact of ABCB1, ABCG2, and CYP3A5 polymorphisms on plasma trough concentrations of apixaban in Japanese patients with atrial fibrillation. *Pharmacogenetics and Genomics*. doi: 10.1097/FPC.0000000000000294.

Ueshima, S., Hira, D., Kimura, Y., Fujii, R., Tomitsuka, C., Yamane, T., et al. (2018). Population pharmacokinetics and pharmacogenomics of apixaban in Japanese adult patients with atrial fibrillation. *British Journal of Clinical Pharmacology* 84, 1301–1312. doi: 10.1111/bcp.13561.
